# Ganglioside Binding Assay: A Complementary Approach for Enhanced Tetanus Toxoid Quality Control

**DOI:** 10.3390/toxins17100500

**Published:** 2025-10-09

**Authors:** Yuki Tanoue, Chie Shitada, Mariko Nakamichi, Naomi Nakamichi, Chiyomi Sakamoto, Hyun Kim, Kohsuke Kumeda, Masaki Ochiai, Susumu Yamaori, Mitsutoshi Senoh, Motohide Takahashi

**Affiliations:** 1 Toxin and Biologicals Research Laboratory, Kumamoto Health Science University, 325, Izumi-machi, Kita-ku, Kumamoto 861-5533, Japan; yuki.tanoue@kaketsuken.org (Y.T.); shitada@kumamoto-hsu.ac.jp (C.S.); sakamoto.c01@kumamoto-hsu.ac.jp (C.S.); 2 The Chemo-Sero-Therapeutic Research Institute (KAKETSUKEN), 8-7, Shinshigai, Chuou-ku, Kumamoto 860-0803, Japan; 3 KM Biologics Co., Ltd., 1-6-1, Okubo, Kita-ku, Kumamoto 860-8568, Japan; nakamichi-ma@kmbiologics.com (M.N.); nakamichi-na@kmbiologics.com (N.N.); kumeda@kmbiologics.com (K.K.); yamaori-su@kmbiologics.com (S.Y.); 4 Department of Bacteriology II, National Institute of Infectious Diseases, 4-7-1, Gakuen, Musashimurayama-shi 208-0011, Tokyo, Japan; hyunk@niid.go.jp (H.K.); senoh@niid.go.jp (M.S.); 5 Center for Quality Management Systems, National Institute of Infectious Diseases, 4-7-1, Gakuen, Musashimurayama-shi 208-0011, Tokyo, Japan; masakio@niid.go.jp

**Keywords:** tetanus toxin, tetanus toxoid, ganglioside binding assay, manufacturing control, quality control test

## Abstract

Vaccine quality control has long relied on animal testing, which involves time, cost, and ethical constraints. This study introduces a ganglioside binding (GB) assay as a complementary in vitro screening tool for tetanus toxoid quality control, which was validated in a single-laboratory environment as a foundational proof-of-concept. The assay reproduces tetanus toxin binding to gangliosides on microplates using simplified procedures. Validation with samples at different inactivation stages showed excellent linearity (0.0002–0.0156 Lf/mL), reproducibility, and a strong correlation with Ramon’s flocculation (R^2^ = 0.999). The assay clearly distinguished between toxins and toxoids, with the toxoid results remaining at control levels. The time-course inactivation samples were consistent with the animal tests: partially inactivated samples (days 1–3) showed significant GB activity (*p* < 0.001) and caused 100% mortality, whereas samples from day 4 onward showed no activity and zero mortality. These findings demonstrate that the GB assay reliably differentiates active toxins from toxoids, which aligns with in vivo outcomes. The practical advantages include a simplified protocol, reduced complexity, and improved efficiency for routine testing of samples. As a complementary screening approach, this single-laboratory validation supports the 3Rs principle by demonstrating the potential for reducing animal use while ensuring quality assurance. Broader applicability requires multicenter validation and cross-reactivity, and multicenter validation is ongoing.

## 1. Introduction

Quality control of biological products has traditionally relied on laboratory animal testing, creating significant challenges for modern vaccine manufacturing, including extended testing timelines, substantial operational costs, and scalability limitations. Recent technological advances have enabled the development of complementary in vitro methods that enhance manufacturing efficiency while supporting ethical research principles through the 3Rs framework (replacement, reduction, and refinement) [1,2,3,4,5]. The implementation of such methods represents a strategic advantage for competitive vaccine production, offering opportunities for enhanced process control, improved testing efficiency, and streamlined quality assurance. Internationally, the quality testing of tetanus toxoids has been standardized, including potency (mouse/guinea pig) and detoxification (guinea pig) tests using laboratory animals [6,7]. Recently, a BINding And CLEavage (BINACLE) assay was developed to detect tetanus toxin using an in vitro method [8,9]. An international collaborative study was conducted by the European Directorate-General for Quality of Medicines and Healthcare to validate the suitability of toxoids for non-toxicity testing [10]. This in vitro test system replicates the functional aspects of tetanus toxin and its in vivo mechanisms of action [11]. Gangliosides serve as essential membrane components and receptors for various neurotoxins, playing crucial roles in neuronal signaling and pathophysiology [12,13,14,15]. Tetanus neurotoxin (TeNT) consists of heavy and light chains linked by disulfide bonds. The toxin enters the cell by binding its heavy chain (H chain) to receptors on neural target cells, and the light chain (L chain) is separated under reducing conditions in inhibitory interneurons. The active L-chain (Fragment C) cleaves synaptobrevin (a SNARE protein), thus interfering with the exocytosis of neurotransmitters from inhibitory interneurons. This blockage of γ-aminobutyric acid (GABA) and glycine release is the direct cause of the physiological effects induced by this toxin. Under normal physiological conditions, GABA inhibits motor neuron activity. Thus, by restricting the release of GABA, tetanus toxin causes spastic paralysis [16]. BINACLE is an in vitro assay that recapitulates the specific two-step TeNT process occurring in vivo. The toxic activity of TeNT was detected by measuring the binding of TeNT to ganglioside N-acetylneuraminyllactosylceramide (GT1b), a receptor for TeNT, as the first step, and the enzymatic activity of the activated L-chain-cleaving synaptobrevin, as the second step. BINACLE has been reported to have a detection sensitivity comparable to that of in vivo tests, and its reproducibility has been verified following the transfer of technology [9]. ELISA-based approaches for tetanus toxoid potency measurement have also demonstrated potential as alternatives to animal testing [17,18]. However, these methods have not yet been implemented in practical applications because of limitations such as prolonged testing durations, limited reagent availability, and challenges in quality control [10,11].

Ganglioside-based binding assays have been previously described for various applications in neurotoxin detection, including antigen ELISA approaches for tetanus vaccine quality control [19,20,21,22]. However, these approaches have not been systematically validated for routine quality control applications in vaccine manufacturing. Although BINACLE provides a comprehensive functional assessment through its dual-step detection of ganglioside binding and enzymatic cleavage activity, this comprehensive approach requires specialized reagents and extended protocols, which may limit its widespread implementation in manufacturing environments. The development of simplified, complementary methods that focus on specific functional aspects may provide practical advantages for routine applications, while acknowledging that comprehensive safety assessments may require multiple complementary approaches.

Therefore, we aimed to develop an assay that would enable simplified quality testing of tetanus toxoids. We constructed a ganglioside binding assay (GB assay) that specifically detects the binding capacity between TeNT and ganglioside and evaluated various samples in collaboration with a vaccine manufacturer. The GB assay methodology can be applied in two complementary modes: direct testing for toxin–toxoid discrimination and, when combined with the reversion test heat treatment, to detect potential toxicity reversion in inadequately inactivated preparations. This study primarily focused on the latter application to develop alternatives to animal-based testing. We validated the consistency of our results with those obtained from guinea pig tests and investigated the potential of this method as an alternative to quality control testing.

Although various in vitro approaches, including cell-based assays, FRET-based detection systems, and nanobioprobe technologies, have been explored for neurotoxin detection [23], their implementation in routine manufacturing environments has been limited by their complexity, reagent availability, and regulatory readiness [24,25]. The GB assay represents a strategic compromise between comprehensive functional assessment (as provided by BINACLE) and practical implementation requirements, which is designed specifically for stepwise integration into existing quality control frameworks through a systematic validation pathway.

## 2. Results

### 2.1. Dose–Response Curves of Toxins and Toxoids in the GB Assay

**Figure 1:**
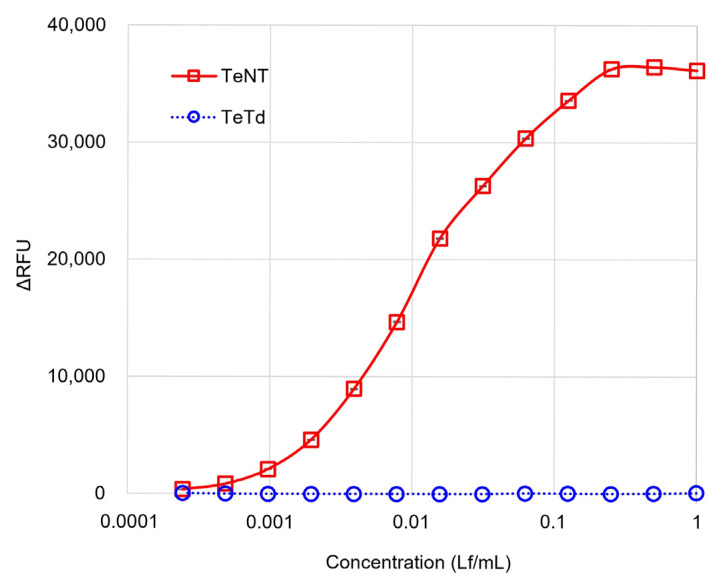
Serial dilutions of TeNT and TeTd were evaluated using the GB assay across a concentration range of 0.0002–1.0 Lf/mL. The *x*-axis represents the test concentration in Lf/mL, and the *y*-axis represents the relative fluorescence units (ΔRFU) calculated by subtracting the background fluorescence signal. TeNT exhibited a typical sigmoidal dose–response curve with a plateau at higher concentrations, whereas TeTd remained within the range of blank controls, demonstrating clear discrimination between active toxins and toxoids. Data are expressed as mean ± SEM from three independent experiments. The error bars are smaller than the symbols because of the high assay reproducibility, with SEM values ranging from 0.25 to 2.88.

TeNTs and TeTd were obtained from a Japanese vaccine manufacturer (KM Biologics Co., Ltd.). These samples were produced using methods equivalent to those approved for vaccine manufacturing and passed the safety tests stipulated in the “Minimum Requirements for Biological Products” [6]. The ganglioside binding capacities of TeNT and TeTd were measured using a GB assay at concentrations ranging from 0.0002 to 1.0 Lf/mL (Figure 1). TeNT plateaued at higher concentrations (0.125–1.0 Lf/mL), resulting in a sigmoidal dose–response curve. In contrast, the TeTd response remained within the range of that of the blank controls, showing no reactivity at higher concentrations (0.125–1.0 Lf/mL). Data are expressed as mean ± SEM from three independent experiments.

### 2.2. Linearity, Specificity, and Stability Tests of GB Assay

**Figure 2:**
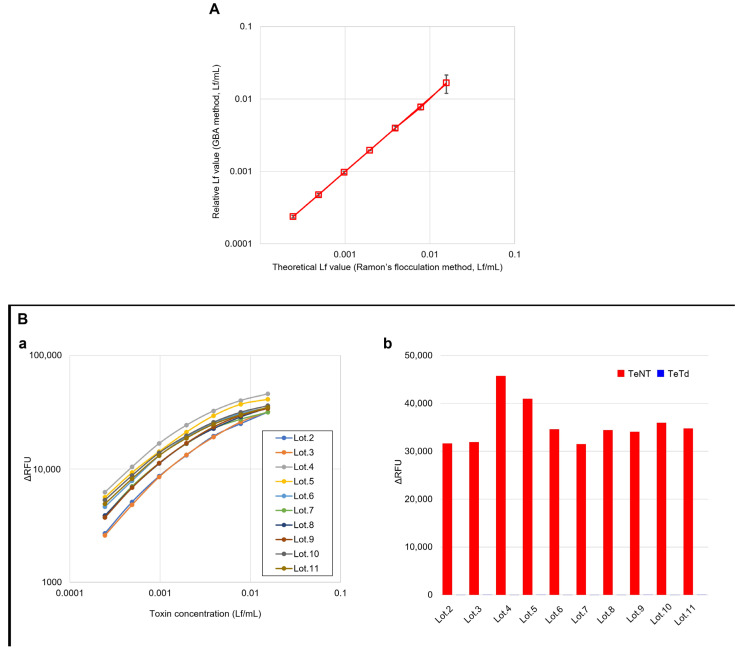

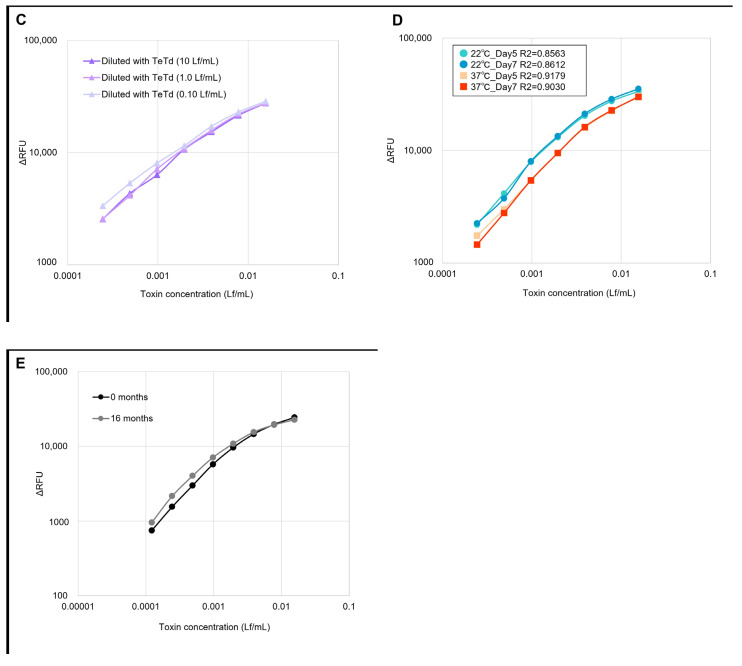

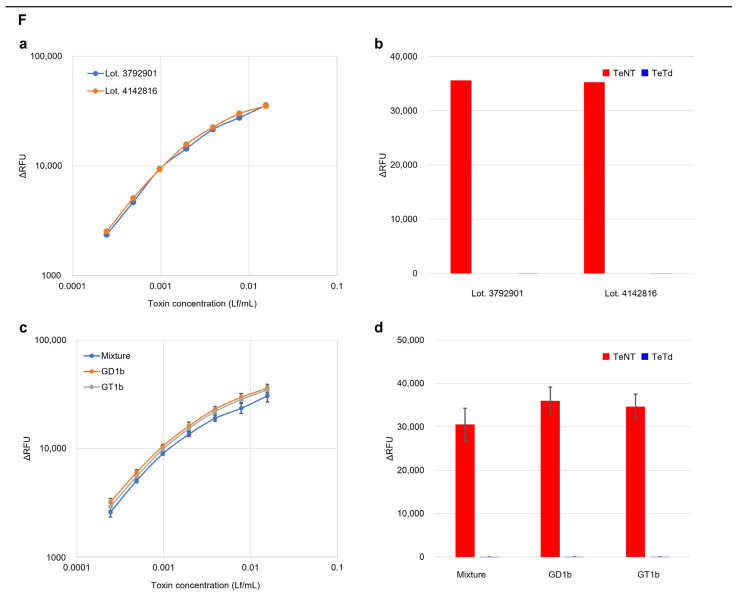
(**A**) Method correlation between the GB assay and Ramon’s flocculation method. *X*-axis: Theoretical Lf values determined using Ramon’s flocculation method. *Y*-axis: Relative Lf values obtained using the GB assay. Strong correlation (R^2^ = 0.999, slope = 1.0) demonstrates quantitative accuracy and method equivalence. (**Ba**) Inter-lot reproducibility assessment using different lots of tetanus toxin preparations across the linear range (0.0002–0.0156 Lf/mL), Fmshowing consistent dose–response patterns across production batches with a coefficient of variation of 8.21% across all lots, and most individual measurements showing high reproducibility of below 10%. (**Bb**) Comparison of TeNT and TeTd responses across different lots at 0.0156 Lf/mL, demonstrating consistent discrimination between active toxins and toxoids. (**C**) Specificity validation: TeNT was serially diluted across the concentration range (0.0002–0.0156 Lf/mL) with TeTd solutions at different concentrations (0.1, 1.0, and 10 Lf/mL), confirming that the presence of toxoid did not interfere with toxin detection throughout the linear range. (**D**) Short-term stability assessment of ganglioside-coated plates across the concentration range (0.0002–0.0156 Lf/mL) after storage at 22 °C or 37 °C for 5 or 7 days. (**E**) Long-term stability validation showing GB assay performance across the concentration range (0.0002–0.0156 Lf/mL) using ganglioside mixture-coated plates stored at 4 °C for 16 months compared to freshly prepared plates, demonstrating good stability for routine quality control applications. (**Fa**,**Fb**) Ganglioside mixture lot-to-lot consistency evaluation showing (**Fa**) dose–response curves across the linear range (0.0002–0.0156 Lf/mL) and (**Fb**) TeNT/TeTd discrimination at 0.0156 Lf/mL across different ganglioside preparation lots. (**Fc**,**Fd**) Comparison of ganglioside coating materials showing (**Fc**) dose–response curves across the concentration range (0.0002–0.0156 Lf/mL) and (**Fd**) TeNT/TeTd discrimination at 0.0156 Lf/mL using ganglioside mixture (Lot. 3792901) versus individual GT1b and GD1b gangliosides. Data in panels (**A**,**Fc**,**Fd**) represent the mean ± SEM of three independent experiments. The data in panels (**Ba**,**Bb**,**C**–**E**,**Fa**,**Fb**) represent the results of individual experiments.

The linearity, specificity, and stability of the GB assay were also evaluated. Linearity testing was performed using seven concentrations (0.0002–0.0156 Lf/mL) of TeNT in 2-fold serial dilutions (*n* = 3) (Figure 2A). The linearity assessment demonstrated a strong correlation between the theoretical Lf concentrations (determined using Ramon’s flocculation method and plotted on the *X*-axis) and the GB assay-calculated Lf values (interpolated from the calibration curve and plotted on the *Y*-axis), with a slope of 1.0 and an intercept showing no significant difference from 0, yielding an R^2^ value of greater than 0.99. Multi-lot validation was performed using ten different lots of TeNT and TeTd to assess inter-lot reproducibility (Figure 2B). All lots demonstrated consistent concentration-dependent response patterns within the linear range (0.0002–0.0156 Lf/mL), with coefficients of variation below 3.3%, confirming the robustness of the GB assay across all production batches. To verify the specificity of the GB assay, toxin samples at seven concentrations (0.0002–0.0156 Lf/mL) were prepared by diluting TeNT with TeTd solutions at different concentrations (10, 1.0, and 0.10 Lf/mL) and measured using the GB assay (Figure 2C). Toxin reactivity was not affected by the concentration of the TeTd solution used for dilution, suggesting that the GB assay specifically detected TeNT. To evaluate the degradation state of the plates, stability tests were conducted by storing them at 22 °C and 37 °C (Figure 2D). Long-term stability evaluation demonstrated that ganglioside-coated plates stored at 4 °C for 16 months maintained robust performance, with coefficient of variation values below 15% compared to freshly prepared plates (Figure 2E), confirming their practical utility in routine quality control applications.

### 2.3. Detection of Toxicity Reversion in Time-Course Inactivation Samples Using GB Assay

**Figure 3:**
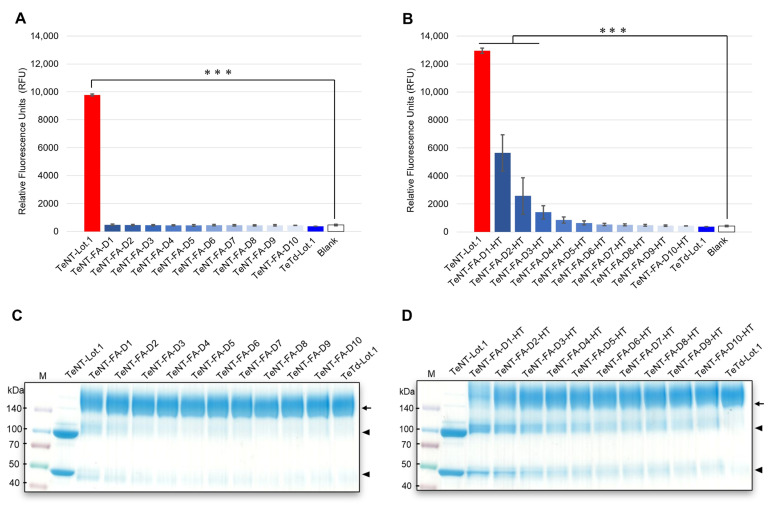
(**A**,**B**) GB assay results using time-course inactivation samples collected daily from days 1 to 10 after the initiation of the inactivation treatment. All samples were tested at a concentration of 0.0156 Lf/mL. TeNT (positive control), TeTd, and Blank (negative control) were included. (**A**) Samples without reversion test heat-treatment. (**B**) Samples subjected to reversion test heat treatment at 37 °C for 20 days. Statistical significance was evaluated using ANOVA with Dunnett’s test compared to that of the blank control group. A Triple asterisk (***) indicates *p* < 0.001. In both graphs, the error bars represent the standard deviation of three independent experiments. (**C**,**D**) SDS-PAGE analysis under reducing conditions. The samples were labeled TeNT-FA-D1 through TeNT-FA-D10, representing time-course inactivation samples from day 1 to day 10; (**C**) Time-course inactivation samples without reversion test heat treatment. (**D**) Samples after reversion test heat treatment at 37 °C for 20 days (labeled with additional -HT). TeNT and TeTd were used as the controls. M: Protein marker (Spectra™ Multicolor Protein Marker [26634]). Arrows indicate cross-linked toxoid (150 kDa), and arrowheads indicate the heavy (100 kDa) and light (50 kDa) chains of the toxin.

A GB assay was performed on time-course-inactivated samples to investigate whether the assay could detect inadequately inactivated toxins. Samples were collected from days 1–10 after initiating TeNT inactivation treatment. Furthermore, to detect potential toxicity reversion, each sample was subjected to heat treatment (37 °C for 20 days) following established reversion testing principles. All samples were standardized to 0.0156 Lf/mL, demonstrating a high response within the linear range. TeTd and most samples without the reversion test heat treatment showed values below the blank value, indicating levels below the quantification limit (Figure 3A). Among the samples subjected to the reversion test heat treatment, those inactivated for shorter periods showed the highest RFU values (Figure 3B). Statistical analysis using Dunnett’s test revealed that in the samples without the reversion test heat treatment, no significant differences were observed between the blank data and any of the inactivation-treated samples. In contrast, among the samples subjected to the reversion test heat treatment, those that were inactivated for 1–2 days showed extremely high RFU values (*p* < 0.0001). The day 3 samples showed high RFU values (*p* = 0.0004), whereas no significant differences were observed for samples collected on or after day 4. Data are expressed as mean ± SD from three independent experiments. Based on these results, prolonged inactivation periods led to the progressive inactivation of the samples exhibited progressive inactivation to levels where toxicity could not be reversed, even with the reversion test heat treatment, demonstrating a loss of ganglioside binding reactivity. The GB assay can detect inadequately inactivated samples with the potential for toxic reversions. As shown in Figure 3A, samples without the reversion test heat treatment showed no significant ganglioside binding activity, suggesting a loss of function during the initial formaldehyde treatment. However, after the reversion test, heat-treated samples from days 1 to 3 of inactivation regained significant ganglioside binding capacity (Figure 3B), indicating the presence of thermolabile crosslinks that could be reversed at physiological temperatures. The differential response patterns between samples without reversion test heat treatment (Figure 3A) and those subjected to reversion test heat treatment (Figure 3B) were particularly evident for samples from days 1 to 3 of inactivation, where the reversion test heat treatment resulted in significantly higher RFU values than those without treatment.

### 2.4. Cross-Linking Status of Time-Course Inactivation Samples

To validate the results obtained from the GB assay, we used sodium dodecyl sulfate-polyacrylamide gel electrophoresis (SDS-PAGE) to examine the degree of formalin cross-linking in time-course inactivated samples collected on days 1–10. All samples, including TeNT and TeTd (used as controls) and time-course inactivated samples, were treated with dithiothreitol (DTT). The extent of crosslinking during the inactivation process was determined by SDS-PAGE and visualized by Coomassie Brilliant Blue staining. A comprehensive quantitative analysis was performed using the ImageJ software (Version 1.54g; National Institutes of Health, Bethesda, MD, USA) to measure the band intensities of all molecular weight species. The reduced TeNT showed separate bands of 100 kDa for the H-chain and 50 kDa for the L-chain, whereas the inactivated TeNT appeared as a distinct band in the 150 kDa region. Time-course inactivation of samples without the reversion test heat treatment demonstrated progressive cross-link formation during the inactivation process, with the 150 kDa band intensity gradually increasing from day 1 to 10, approaching TeTd levels (Figure 3C). Samples subjected to the reversion test heat treatment are shown (Figure 3D) as 100 kDa and 50 kDa bands, which were markedly stronger with short inactivation periods (days 1–3) than with TeTd treatment. ImageJ quantitative analysis revealed highly significant increases in DTT-reducible bands for day 1–3 samples after reversion test heat treatment, with 36.4–64.8% increases compared to samples without reversion test heat treatment (*p* < 0.001). This provides direct molecular evidence of thermolabile crosslinking during the early stages of the inactivation process. The intensity of these bands gradually decreased with increasing inactivation time, suggesting reduced susceptibility to DTT.

### 2.5. Guinea Pig Tests Using Time-Course Inactivation Samples

To investigate the time-course reactions of inactivated samples in animals, each sample was administered to guinea pigs at different doses: 500 Lf/head for samples without reversion test heat treatment and 40 Lf/head for samples subjected to reversion test heat treatment. Observations were conducted over a standardized 21-day period. The survival rates, symptoms, and changes in body weight of four guinea pigs in each group were monitored daily. Observations were conducted 21 days post-injection for each sample.

**Figure 4:**
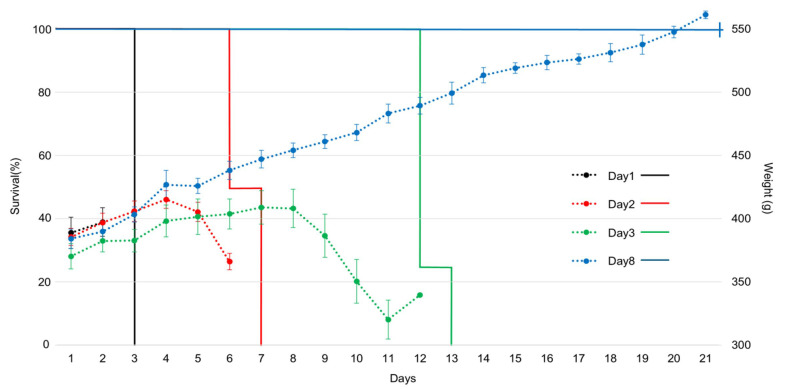
Kaplan–Meier survival curves (solid lines) and mean body weight changes (dashed lines) of guinea pigs injected with inadequately inactivated toxin samples without the reversion test heat treatment. The data are shown for guinea pigs injected with the samples on days 1, 3, and 8 after inactivation. Error bars indicate the standard error of the mean (*n* = 4 guinea pigs per group). Guinea pigs injected with samples from days 1 to 3 tended to die after experiencing rapid weight loss.

For samples without the reversion test heat treatment, guinea pigs injected with TeNTs inactivated for 1 d died on day 3 without showing any symptoms after injection, indicating a rapid reversion to toxicity (Table 1 and Table 2). Guinea pigs injected with inactivated TeNT for 2 days showed one of two patterns: sudden death after an asymptomatic period or death with progressive paralysis. Guinea pigs that received inactivated TeNT for 3 days exhibited progressive paralysis symptoms for 4–6 days before death. In contrast, in samples subjected to the reversion test heat treatment, the onset of symptoms and mortality occurred more rapidly. Guinea pigs injected with inactivated TeNT for 1 d developed severe paralysis symptoms on the first day and died on the second day post-injection. TeNT inactivation for 2 days caused mild paralytic symptoms on the second day post-injection, which progressed to death by days 3–4 after injection. Guinea pigs receiving inactivated TeNT for 3 days showed a delayed onset of symptoms starting on day 6 post-injection, with rapid progression to severe paralysis and death by day 8. For samples subjected to inactivation treatment for four days or longer under our experimental conditions, no symptoms or deaths were observed in any guinea pig, regardless of the heat treatment during reversion testing. Body weight was measured daily after sample administration (Figure 4). Guinea pigs show rapid weight loss 2–3 days before death.

## 3. Discussion

The development of the GB assay represents a foundational step in a systematic approach to reduce animal testing for tetanus toxoid quality control. Rather than attempting to immediately replace established methods, this study provides proof-of-concept validation within a controlled, single-laboratory environment, positioning the GB assay as a complementary screening tool that provides the essential groundwork for broader implementation. This strategic approach aligns with regulatory science principles, wherein stepwise validation is recognized as the standard pathway for the introduction of alternative testing methods.

The GB assay complements existing comprehensive approaches, particularly the well-established BINACLE assay, by specifically focusing on the ganglioside binding function of tetanus toxin. While BINACLE’s dual-step detection (binding + enzymatic cleavage) provides comprehensive toxin characterization essential for detailed safety assessment, the GB assay offers a focused approach suitable for specific quality control scenarios in which rapid binding capacity assessment is prioritized. We acknowledge that measuring ganglioside binding alone provides an incomplete functional assessment compared to methods that evaluate both binding and enzymatic activity. However, for certain routine applications, this focused approach may offer practical advantages, including simplified implementation and reduced complexity.

The GB assay is a flexible alternative methodology that can be implemented according to different quality control requirements. The core ganglioside binding detection method remained constant, whereas the sample preparation protocol (with or without reversion test heat treatment) was selected based on the testing objectives on the study. For routine quality control of properly manufactured toxoids, a direct GB assay without heat treatment enables rapid discrimination between toxins and toxoids. However, for a comprehensive safety evaluation, particularly during process validation or investigation of potential quality issues, the GB assay combined with the reversion test heat treatment offers enhanced sensitivity for detecting inadequately inactivated preparations with the potential for toxic reversion. This dual-application approach aligns with regulatory flexibility, allowing the method to adapt to varying quality control requirements while maintaining the core simplicity and reliability of the ganglioside-binding-detection principle.

Our single-laboratory validation with KM Biologics represents the essential first phase of the systematic validation pathway recommended for alternative testing methods. This controlled environment allowed us to establish fundamental assay parameters, optimize protocols, and demonstrate proof-of-concept correlations with existing methods (R^2^ = 0.999). Such single-site validation is a prerequisite for multicenter studies in regulatory science, providing foundational data necessary for broader collaborative validation efforts.

The use of animal testing for correlation analysis, while seemingly contradictory to our goal of reducing animal use, represents a strategic “investment” in animal welfare by using a limited number of animals to establish robust alternatives that could prevent extensive animal use in the future. This approach aligns with the 3Rs principle of “reduction” as an interim step toward eventual “replacement,” following successful precedents in vaccine development, where initial validation required animal correlation before achieving regulatory acceptance.

Based on current regulatory trends and the successful implementation of alternative testing, we propose a three-phase implementation pathway.

- Phase 1 (Current study): Single-laboratory validation and correlation with existing methods;
- Phase 2 (Proposed): Multi-center collaborative studies and regulatory consultation;
- Phase 3 (Future): Regulatory acceptance and potential acceptance for routine implementation.

This study completed Phase 1, providing validated methodology and correlation data necessary for advancing broader validation studies.

Although the current single-laboratory validation provides essential proof-of-concept data, comprehensive implementation requires addressing several key validation requirements in the future. Cross-reactivity evaluation with other clostridial toxins (*C. botulinum*, *C. novyi*, and *C. perfringens*) is currently in progress, with toxin procurement and regulatory approvals underway. Multi-center validation studies involving independent laboratories are planned to establish inter-laboratory reproducibility parameters and standardized protocols.

The reversion test heat treatment of the samples revealed that the GB assay could detect inadequately inactivated toxin activity with higher sensitivity than current animal testing methods, particularly for identifying partially inactivated preparations. This enhanced detection capability manifests in several key aspects, enabling detailed monitoring of the inactivation process: (1) the ability to detect statistically significant differences between samples at consecutive time points during inactivation (days 1–10), as demonstrated by our time-course analysis; (2) the capacity to generate reproducible measurements with a low coefficient of variation (<15%) that correlates with the degree of inactivation; (3) the sensitivity to detect subtle changes in binding capacity that correspond to different stages of cross-linking, as confirmed by SDS-PAGE analysis; (4) the capability to detect potential toxicity at concentration levels below the threshold required to produce symptoms in animal models. The observed correlation between the GB assay results and animal survival rates, combined with the molecular evidence from SDS-PAGE analysis demonstrating progressive cross-linking, suggests that our method provides more detailed monitoring of the inactivation process and offers significant advantages for the safety assessment of biopharmaceuticals. This enhanced detection capability was demonstrated using samples with and without reversion test heat treatment (37 °C for 20 days), conditions that follow established regulatory principles for detecting thermolabile cross-links that could potentially restore toxicity under physiological conditions. This precision was further enhanced by the ability of the assay to detect formaldehyde-induced Schiff base linkage formation and its reversibility under thermal stress conditions. Unlike animal testing, which primarily provides binary outcomes (toxic/non-toxic), our GB assay provides continuous quantitative data across the inactivation timeline, enabling a more granular safety assessment of products.

The molecular mechanisms underlying the differential detection capabilities of heated and non-heated samples likely involve the formation of reversible crosslinks during the early stages of formaldehyde inactivation. During the initial inactivation period, formaldehyde forms Schiff base linkages [26,27] that temporarily mask the ganglioside binding pocket of the toxin without permanently inactivating it [28]. Heat treatment at a physiological temperature (37 °C) can reverse these unstable bonds, restoring the binding capacity of the toxin and revealing its potential to reverse toxicity. Although both regular and heat-treated materials can cause mortality in animal tests, heat treatment accelerates the reversion to toxicity in marginally inactivated preparations, providing an early warning system for detecting inadequately inactivated samples. This approach aligns with established quality control principles, wherein toxicity reversion testing reveals potential long-term stability issues, thereby allowing a more comprehensive safety assessment. The GB assay, when combined with the reversion test heat treatment, serves as an effective in vitro method for detecting thermally induced toxicity reversion, offering a safer and more practical alternative to extensive animal testing for the toxicity reversion assessment of chemicals. This explains why samples that appeared adequately inactivated under pre-reversion test conditions (Figure 3A) showed significant binding activity after heat treatment (Figure 3B), particularly for samples with shorter inactivation periods (days 1–3). As the inactivation time progressed beyond day 4, more stable crosslinks were formed, making inactivation irreversible, even under reversion test conditions.

Another key finding was the differential detection of toxin activity in samples with and without heat treatment during the reversion test. Although inadequately inactivated samples without the reversion test heat treatment showed no detectable activity in the GB assay, they caused mortality in animal tests. These findings suggest that heat treatment-based toxicity reversion testing is essential, even for in vitro methods that serve as alternatives to animal testing, as it reveals potential toxicity reversions that may be undetected using standard GB assays.

Behrensdorf-Nicol et al. (2008) showed that tetanus toxoids inactivated with formaldehyde retained a substantial degree of residual vesicle-associated membrane protein 2 (VAMP2) cleavage activity [29]. Although the toxoid batches used in these experiments were obtained from several vaccine manufacturers, no in vivo toxicity was detected in the animal tests. This finding demonstrates that intact ganglioside binding capacity is essential for tetanus toxicity expression; without this initial receptor binding, the toxin cannot enter cells to exert its enzymatic activity, regardless of how well it is preserved. This mechanistic understanding explains why toxins with impaired ganglioside binding cannot cause toxicity in vivo, confirming that measuring enzymatic activity after cellular entry may not provide highly informative results for inactivation testing.

Recent work by Behrensdorf-Nicol and Krämer (2019) reported that tetanus toxin undergoes molecular degradation under reversion test conditions (37 °C, 20 days) when evaluated using the BINACLE assay [30]. While these studies demonstrated structural changes at the molecular level, our GB assay results suggest that ganglioside binding capacity can be restored in inadequately inactivated samples under reversion test conditions. This indicates that the critical receptor recognition domain recovers its functional activity when thermolabile Schiff base cross-links are disrupted. The correlation between our GB assay findings and animal test outcomes supports this interpretation, as heat-treated samples from the early inactivation stages (days 1–3) regained sufficient binding activity to cause guinea pig death. This highlights the importance of assessing functional binding activity rather than relying solely on molecular integrity for the comprehensive safety evaluation of tetanus toxoid preparations. However, a direct comparison between these studies requires careful consideration of the methodological differences. BINACLE investigations utilized pure tetanus toxin and commercially available toxoid preparations that had completed the full manufacturing process, whereas our study examined formaldehyde-treated samples collected at various time points during incomplete inactivation (days 1–10). In addition, the experimental protocols, sample preparation methods, and detection endpoints differ substantially between these two approaches. These methodological distinctions may contribute to the contrasting observations regarding molecular integrity and functional binding activity under reversion test conditions. These differences underscore the importance of employing complementary analytical approaches to comprehensively evaluate the complex biochemical changes that occur during tetanus toxin inactivation and the potential reversion processes.

We recognize that the severe consequences of releasing tetanus toxoid-containing active toxins necessitate a comprehensive safety assessment, which ideally includes an evaluation of both binding and enzymatic activity. While our data suggest that ganglioside binding capacity provides sensitive detection of inadequately inactivated preparations, we acknowledge that this represents only one aspect of tetanus toxin function. The BINACLE assay’s comprehensive evaluation of both binding and enzymatic activity provides a more complete functional assessment, which may be essential for definitive safety evaluation. Our GB assay should be considered a complementary screening tool that may be particularly useful for routine screening applications, with comprehensive methods such as BINACLE potentially reserved for confirmatory testing and detailed characterization.

The GB assay, particularly when combined with reversion test heat treatment, demonstrated strong concordance with animal testing outcomes in identifying inadequately inactivated preparations with the potential for toxic reversion. For routine quality control purposes, we propose the GB assay as a complementary screening alternative to animal testing, with enzymatic activity testing potentially reserved for the validation stages or investigation of anomalous results. This approach balances comprehensive safety assessments with practical implementation considerations while adhering to the 3Rs of animal welfare. To further enhance the comprehensive safety assessment, we are currently developing a complementary Fluorescence Resonance Energy Transfer (FRET) assay to measure enzymatic cleavage activity. This developmental work, which will be used in conjunction with our established GB assay, utilizes FRET substrates containing VAMP2 cleavage sequences specific to the tetanus toxin, and we successfully confirmed detectable cleavage activity in preliminary studies. Although sensitivity optimization remains a technical challenge, this combined approach may provide both binding and enzymatic assessment capabilities within a unified in vitro testing framework.

According to WHO manuals and various national standards, conventional animal-based toxicity reversion testing requires pre-incubation of samples at 37 ± 1 °C for 42 days before animal testing. Our GB assay protocol, following the Japanese Minimum Requirements for Biological Products, used a shorter pre-incubation period (20 days vs. 42 days), which could potentially accelerate batch release compared to standard procedures. The pre-incubation step is a regulatory requirement for reversion testing, regardless of the detection method used, and our GB assay complies with this requirement while offering reduced animal use and more sensitive detection of inadequately inactivated samples.

Furthermore, our data demonstrate that the GB assay can be implemented as an alternative to animal testing in two complementary modes: (1) a direct GB assay without heat treatment for discriminating between toxins and properly inactivated toxoids; (2) a GB assay with heat treatment for comprehensive safety evaluation during production validation or investigation of potential toxicity reversion. As regulatory frameworks evolve, including considerations by organizations such as the European Pharmacopoeia to eliminate reversion testing for tetanus toxoid and WHO recommendations for removing innocuity tests from biological product requirements [31], the GB assay offers flexibility to adapt to these changing requirements.

This study presents several characteristics that define its role as foundational research in a broader validation program. First, validation was conducted within a single manufacturing environment (KM Biologics Co., Ltd.), which represents the appropriate first step for establishing assay parameters and demonstrating proof of concept before a multicenter expansion. This controlled approach allowed for the systematic optimization of protocols and thorough characterization of assay performance, providing robust baseline data essential for subsequent collaborative studies.

Second, although we aimed to develop alternatives to animal testing, our validation necessarily relied on guinea pig studies for correlation analysis, representing the strategic use of limited animal testing to establish robust alternatives for future widespread implementation. This approach follows established precedents in regulatory science, where initial animal correlation studies are accepted as necessary investments to validate alternatives that will ultimately prevent the use of animal in routine applications.

Third, the GB assay measures ganglioside binding capacity without assessing enzymatic activity, positioning it as a focused screening tool rather than a comprehensive replacement for other assays. This targeted approach offers distinct advantages in terms of simplicity and implementation while acknowledging that comprehensive safety assessments may require integration with complementary methods. Future development phases could incorporate enzymatic assays to create a complete in vitro testing framework based on the validated binding detection established in this study.

Regarding regulatory acceptance, while in vitro alternatives are increasingly supported by regulatory frameworks and consistency-based approaches are being implemented [32], the GB assay should currently be considered a proof-of-concept building block for future regulatory packages, rather than an immediate replacement for established methods. The strong correlation data (R^2^ = 0.999) and systematic validation approach provide the foundational evidence required for advancing multicenter collaborative studies and regulatory consultation phases [33,34].

Various methods have been developed to detect clostridial neurotoxins, including tetanus toxin, botulinum toxins, and their toxoids, using monoclonal antibody-based approaches [35], nanobioprobe technologies, cell-based assays, and fluorescent immunochromatography [36,37,38]. Although these in vitro methods are potential alternatives to conventional methods, significant challenges remain regarding their reproducibility and regulatory validation. The GB assay technology has been successfully transferred to collaborating institutions, including KM Biologics Co., a vaccine manufacturing company, and the National Institute of Infectious Diseases. Although there were differences in the measurement values owing to differences in the measurement equipment, the reproducibility of the GB assay was confirmed. Further studies are needed to expand this technology to additional domestic manufacturers and to verify the precision of the GB assay across different manufacturing processes and product lots.

### Study Limitations

This proof-of-concept study has several important limitations that define its current scope.

- Single-laboratory validation environment: All validations were conducted within a controlled setting involving Kumamoto University of Health Sciences and KM Biologics Co., Ltd., representing Phase 1 validation that requires subsequent demonstration of transferability to independent laboratories.
- Cross-reactivity assessment pending: Evaluation against other clostridial neurotoxins (*C. botulinum*, *C. novyi*, and *C. perfringens*) is currently in progress, with toxin procurement and regulatory approvals underway.
- Inter-laboratory reproducibility had not yet been established: Although technology transfer to collaborating institutions has been initiated, formal multicenter validation studies are required to establish reproducibility parameters across different manufacturing processes and laboratory settings.
- Regulatory acceptance is contingent on future validation: Implementation as a routine testing method depends on the successful completion of multicenter validation and regulatory consultation phases.

## 4. Conclusions

We developed and validated a GB assay as a complementary screening tool that demonstrated a strong correlation (R^2^ = 0.999) with established methods within a controlled single-laboratory environment, completing the essential first phase of systematic validation toward the potential reduction in animal testing in tetanus toxoid quality control. This proof-of-concept study lays the foundation for broader multicenter validation studies. Although this represents only the initial step in a comprehensive validation pathway, the results provide robust proof-of-concept data supporting the potential of this assay as a practical screening tool for future portfolios of animal testing alternatives.

The practical advantages of this methodology’s including simplified procedures, reduced reagent requirements, and compatibility with existing quality control workflows, combined with strong analytical performance, support its advancement to multicenter validation studies. The GB assay should be considered a complementary screening tool within a portfolio of quality control methods rather than an immediate replacement for established animal testing methods. Regulatory acceptance requires successful multicenter validation and demonstration of interlaboratory reproducibility. Future implementation requires collaborative validation across multiple laboratories, cross-reactivity evaluation with other clostridial toxins, and potential integration with complementary enzymatic assays, such as the planned FRET-based system. Recent surveys indicate a growing industry interest in integrating 3Rs approaches into WHO guidelines for biological products [39], supporting the strategic importance of developing proof-of-concept alternatives, such as the GB assay. This foundational study provides validated methodology and correlation data necessary for pursuing the next critical steps toward a meaningful reduction in animal testing requirements.

## 5. Materials and Methods

### 5.1. Tetanus Toxin and Tetanus Toxoid

Purified tetanus toxin (TeNT) and tetanus toxoid (TeTd) were obtained from KM Biologics Co., Ltd. (Kumamoto, Japan). The representative lots used for the primary experiments were TeNT Lot. 1 (720 Lf/mL, 1.825 mg/mL) and TeTd Lot. 1 (580 Lf/mL, 1.575 mg/mL). For method validation and inter-lot reproducibility assessment, multiple lots were used: TeNT Lot. 2–11 (range: 540–990 Lf/mL, 1.381–2.150 mg/mL) and TeTd Lot. 2–11 (range: 580–825 Lf/mL, 1.469–2.044 mg/mL). The specific activity of TeNT Lot. 1 was determined to be 6.5 × 10^7^ LD50/mg using the mouse challenge test. TeNT and TeTd preparations were aliquoted into single-use portions and stored at −80 °C, with aliquots thawed before each use.

### 5.2. Measurement of Lf Value

Lf was measured according to the method described in the World Health Organization manual for the control of vaccine production, which involves the detection of the complex formed between the antigen and antibody [3,9,10]. Lf represents the amount of toxins or toxoids that form a visible precipitate at an optimal ratio when mixed with an International Unit of the antitoxin. The antitoxin was diluted in saline to a concentration of 100 U/mL, and equal volumes were added to each of the test tubes. Saline was then added to achieve a final volume of 1 mL. Samples were prepared such that the flocculation reaction with the antitoxin occurred under optimal conditions, and 1 mL was added to each tube. The samples were placed in a 50 °C thermostatic bath, and one-third of each tube was immersed. The appearance of the flocs was monitored using natural convection. The Lf value was calculated using the dilution factor of the sample in the first tube.

### 5.3. Toxin Samples with Variable Inactivation Periods

The inactivation procedure was adapted from the manufacturing process used by KM Biologics Co., Ltd. Tetanus toxoid production was scaled to laboratory conditions to ensure the industrial relevance of the experimental conditions. The TeNT working solution was prepared at a theoretical concentration of 500 Lf/mL by diluting the stock solution with phosphate buffer (0.1 M). Two amino acids, the specific types of which were provided by the manufacturer, were added as stabilizers to the medium. The solution was then filtered through a 0.2 µm filter and transferred to a stainless-steel container. After formalin was added, the mixture was incubated at 35 °C for 10 days. Time-course inactivation samples were prepared by daily sampling for 10-day period. Formalin was subsequently removed from each sample by dialysis against 13.3 mM phosphate buffer (pH 7.2). The Lf value of each final dialyzed sample was determined for characterization using the flocculation method (Section 2.2). However, to evaluate the GB assay, all samples were standardized based on their initial theoretical concentrations. To ensure a fair comparison of residual toxicity, all time-course inactivated samples were diluted to a standardized concentration of 0.0156 Lf/mL. This dilution factor was calculated based on an initial theoretical concentration of 500 Lf/mL to ensure that an equivalent amount of the starting material was assayed at each time point.

### 5.4. Ganglioside Binding (GB) Assay

#### 5.4.1. Ganglioside-Coated Plate

FluoroNunc™ Black 96-Well Immuno Plates MaxiSorp (Thermo Fisher Scientific, Waltham, MA, USA) were used for the assays. A ganglioside mixture (Lot. 3792901; ammonium salt, bovine brain-derived, 25 mg; Merck, Tokyo, Japan) was prepared at a concentration of 500 μg/mL in methanol and diluted to the desired concentrations with phosphate-buffered saline (PBS) containing 0.1% sodium azide (pH 7.4). Alternative ganglioside sources showing equivalent performance (as demonstrated in Figure 2F) may be considered for technology transfer after appropriate verification. A volume of 200 μL (at 2 μg/well) was added to each well of the plate and incubated overnight at 4 °C. The ganglioside preparation was removed, and 320 μL of blocking buffer (1% bovine serum albumin (BSA), 0.1% sodium azide, 5% lactose, and 5% sucrose in PBS) was dispensed per well and incubated at 25 °C overnight. The blocking buffer was removed, and the plates were dried in a desiccator until the humidity was less than 35%. The plates were vacuum-packed in aluminum laminates with a desiccant and stored at 4 °C. The prepared plates were suitable for use for up to one year from the date of preparation.

#### 5.4.2. GB Assay

The samples (TeNT or TeTd) were diluted to the measurement concentration with 1% BSA in phosphate buffer (PB), and 200 μL of each diluted sample was added to each well and incubated overnight at 4 °C. The plates were washed thrice with 9.6 mM phosphate buffer (PB; pH 7.1) containing 1% bovine serum albumin (BSA). Anti-TeNT polyclonal antibodies were purified using affinity purification of serum obtained from rabbits highly immunized with tetanus-adsorbed toxoid, using Complete Freund’s adjuvant for the primary immunization and Incomplete Freund’s adjuvant for booster injections. Purified antibodies were labeled using a peroxidase-labeling Kit-NH_2_ (Dojindo, Kumamoto, Japan). The labeled antibody (200 ng/mL) was diluted 100-fold in a conjugate buffer consisting of 1/2500 ProClin 300 (Sigma-Aldrich, St. Louis, MO, USA), 0.005% bromocresol purple (TCI, Tokyo, Japan), 1 mM MgCl_2_, and 1% BSA in PB, and 200 µL was added to each well and incubated at 4 °C overnight. The plates were washed thrice with 0.05% Tween 20 in PB. Subsequently, 200 µL of substrate solution from the QuantaBlu™ Fluorogenic Peroxidase Substrate Kit (Thermo Fisher Scientific, Tokyo, Japan) was added to each well and incubated at 37 °C for 30 min. After incubation, 100 µL of stop solution was added to the same kit. Relative fluorescence units (RFU) were measured using an Infinite^®^ 200 PRO (TECAN, Maulbronn, Switzerland), and a calibration curve was obtained. The measurement conditions were as follows: fluorescence intensity, wavelength (excitation: 325 nm emission: 420 nm), mode (top), Z-position (Manual: 21,875 μm), read (emission frequency: 25 times settling time: 15 ms), gain (80), and integration (integration time: 20 μs). When necessary, the effective active toxin concentration in the inactivated samples was estimated by interpolating the measured RFU values onto the linear portion of the calibration curve established using known toxin concentrations.

#### 5.4.3. Method Validation

For the linearity assessment, the TeNT stock solution was serially diluted 2-fold to prepare seven concentrations ranging from 0.0002 to 0.0156 Lf/mL, and each concentration was measured in triplicate using the GB assay protocol described in Section 5.4.2 of this manuscript. The measured RFU values were interpolated onto the linear portion of the calibration curve to obtain the corresponding Lf/mL concentrations, which were then compared with the theoretical concentrations calculated from Ramon’s flocculation-determined stock concentrations and dilution factors.

The inter-lot reproducibility was assessed using TeNT Lot. 2–11 and TeTd Lot. 2–11 prepared at concentrations ranging from 0.0002 to 0.0156 Lf/mL, with additional measurements performed at 0.0156 Lf/mL for a direct comparison.

Specificity validation was conducted by diluting the TeNT stock using TeTd solutions (0.1, 1.0, and 10 Lf/mL) instead of the buffer to prepare concentrations ranging from 0.0002 to 0.0156 Lf/mL, which were then measured using the standard GB assay protocol.

For stability testing, ganglioside-coated plates were stored under different conditions (22 °C and 37 °C for 5–7 days, or 4 °C for 16 months) and tested using TeNT concentrations ranging from 0.0002 to 0.0156 Lf/mL.

Ganglioside preparation was compared using plates prepared with different ganglioside mixtures (Lot. 3792901, Lot. 4142816) and individual gangliosides, including GD1b (A01896, bovine-derived disodium salt, 1 mg; AdipoGen, San Diego, CA, USA) and GT1b (A01796, bovine-derived disodium salt, 1 mg; AdipoGen, San Diego, CA, USA) following the protocol described in Section 5.4.1, and then tested with TeNT concentrations ranging from 0.0002 to 0.0156 Lf/mL.

### 5.5. Animal Tests

Animal tests were conducted according to the tetanus toxoid detoxification test described in the “Minimum Requirements for Biological Products” [2]. Four female SPF guinea pigs (Hartley strain) (Japan SLC, Shizuoka, Japan) weighing approximately 300–400 g were used for each sample. Time-course inactivated samples were diluted in 0.017 mol/L phosphate-buffered sodium chloride solution (pH 7.0). Samples from days 1–5 and 8 of the inactivation time course (5 mL) were subcutaneously injected into the right medial thigh of guinea pigs. Samples without reversion test heat treatment were administered 500 Lf/head, whereas samples subjected to reversion test heat treatment (37 °C for 20 days) were administered 40 Lf/head. The animals were observed for at least 21 days for toxin-induced symptoms, including death, convulsions, paralysis, and significant weight loss. All animal experiments conducted in this study were approved by the Animal Experimentation Committee (Approval No.: 23-02, R240623-1C) and were conducted in strict compliance with the guidelines on animal welfare of the Kumamoto University of Health Sciences and KM Biologics Co., Ltd.

### 5.6. SDS-PAGE Analysis

Samples were mixed in equal volumes with a sample buffer containing 100 mM dithiothreitol and heat-treated at 95 °C for 5 min. Subsequently, 0.75 Lf (approximately 2 µg protein) was loaded onto a 5–20% polyacrylamide gel (ATTO, Tokyo, Japan) in an electrophoresis chamber for SDS-PAGE. After electrophoresis at 100 V for 60 min, the gels were stained with Coomassie protein (Abcam, Cambridge, UK) in the distilled water. Quantitative analysis of the band intensities was performed using the ImageJ software (National Institutes of Health, Bethesda, MD, USA). The band intensities of 150 kDa (cross-linked), 100 kDa (heavy chain), and 50 kDa (light chain) were measured and normalized to the background levels for statistical comparison.

### 5.7. Statistical Analysis

Analysis of variance (ANOVA) was used to compare the results of the toxoid samples prepared using various inactivation periods, and multiple comparisons among the sample groups were conducted using Dunnett’s test. Statistical significance was set at *p* < 0.05. For dose–response curves and animal studies, the results are presented as mean ± SEM. For time-course inactivation sample analysis, the results are expressed as mean ± SD.

## Figures and Tables

**Table 1:** Summary of symptoms and survival in guinea pigs challenged with time-course inactivation samples.

Heat Treatment of Samples	Preparation Volume	Injection Dose		Observations		Samples: Days After Inactivation Initiation					
	(Lf/mL)	(mL)	(Lf/Head)			Day 1	Day 2	Day 3	Day 4	Day 5	Day 8
-	100	5	500	Time to onset	Paralysis	ND	6	6	ND	ND	ND
					Death	3	6	12	ND	ND	ND
				Survival/Number of Guinea Pig		0/4	0/4	0/4	4/4	4/4	4/4
37 °C 20 days	8	5	40	Time to onset	Paralysis	1	2	6	ND	ND	ND
					Death	2	2	8	ND	ND	ND
				Survival/Number of Guinea Pig		0/4	0/4	0/4	4/4	4/4	4/4

ND; Not Detected.

**Table 2:** Daily observation of symptoms in guinea pigs after challenge with time-course inactivation samples.

Samples	In Vitro Reversion Test	Injection Dose (Lf/Head)	Animal No.	Days Post Challenge												
				1	2	3	4	5	6	7	8	9	10	11	12	13
Day 1	-	500	1	-	-	d										
			2	-	-	d										
			3	-	-	d										
			4	-	-	d										
	+	40	5	+++	d											
			6	+++	d											
			7	+++	d											
			8	+++	d											
Day 2	-	500	9	-	-	-	-	-	d							
			10	-	-	-	-	-	d							
			11	-	-	-	-	-	++	d						
			12	-	-	-	-	-	+	d						
	+	40	13	-	+	d										
			14	-	-	d										
			15	-	+	+++	d									
			16	-	-	d										
Day 3	-	500	17	-	-	-	-	-	+	+	+	+	+	+++	d	
			18	-	-	-	-	-	+	-	+	+	+	+++	d	
			19	-	-	-	-	-	-	-	+	+	+	+++	+++	d
			20	-	-	-	-	-	-	-	+	+	+	+++	d	
	+	40	21	-	-	-	-	-	-	+	++++	d				
			22	-	-	-	-	-	-	+	++++	d				
			23	-	-	-	-	-	+	+	d					
			24	-	-	-	-	-	+	+	++++	d				

Symptoms severity: -, no symptoms; +, mild paralysis; ++, moderate paralysis; +++, severe paralysis; ++++, very severe paralysis. d, death; ND, not detected.

## Data Availability

The data presented in this study are available upon request from the corresponding author. Standard operating procedures (SOPs), acceptance criteria, and validation protocols may be shared to support planned inter-laboratory validation studies, subject to appropriate collaborative agreements.
